# Complications of Intrathecal Chemotherapy in Adults: Single-Institution Experience in 109 Consecutive Patients

**DOI:** 10.1155/2019/4047617

**Published:** 2019-05-02

**Authors:** Diana M. Byrnes, Fernando Vargas, Christopher Dermarkarian, Ryan Kahn, Deukwoo Kwon, Judith Hurley, Jonathan H. Schatz

**Affiliations:** ^1^Hematology-Oncology Fellowship Program, Department of Medicine, Jackson Memorial Hospital, USA; ^2^Miami Cancer Institute, Baptist Health South Florida, USA; ^3^University of Miami Miller School of Medicine, USA; ^4^Biostatistics and Bioinformatics Core, USA; ^5^Sylvester Comprehensive Cancer Center, USA; ^6^Division of Hematology, Department of Medicine, University of Miami Miller School of Medicine, Miami, FL, USA

## Abstract

Acute lymphoblastic leukemia and other aggressive lymphoid malignancies like Burkitt leukemia/lymphoma have high incidence of central nervous system (CNS) involvement. Various solid tumors, most notably breast cancer, can also metastasize into the CNS as a late stage complication causing devastating effects. Intrathecal (IT) chemotherapy consisting of methotrexate, cytarabine, or the two in combination is frequently used for the prophylaxis and treatment of CNS metastasis. Because of the high toxicity of these chemotherapeutic agents, however, their side effect profiles are potentially catastrophic. The incidence of neurotoxicity secondary to IT chemotherapy is well defined in the pediatric literature but is poorly reported in adults. Here, we investigated the incidence of neurologic and nonneurologic side effects secondary to IT chemotherapy in 109 consecutive adult patients over a two-year time period at hospitals associated with our institution. Of 355 IT chemotherapy treatments received by these patients, 11 (3.10%) resulted in paresthesias or paralysis, which we defined as significant neurologic events in our analysis. We also examined minor events that arose after IT chemotherapy, including back pain, headache, fever, vomiting, and asthenia. At least one of these occurred after 30.70% of IT chemotherapy doses. Clinicians involved in the care of patients receiving IT chemotherapy should be aware of these findings and consider treatment options lower rate of neurotoxicity such as high-dose systemic methotrexate.

## 1. Introduction

Advances in the treatment of many hematological and solid tumor malignancies have improved disease-free survival rates. Unfortunately, such improvements have come with increased frequency of relapses in the leptomeninges or CNS parenchyma, most commonly in aggressive lymphoid malignancies such as acute lymphoblastic leukemia (ALL), Burkitt lymphoma/leukemia, and lymphoblastic lymphoma [1–4]. Leptomeningeal metastasis can also complicate solid tumors, breast carcinoma being the most commonly associated [5]. Several treatments have been developed to target malignant cells in the CNS to prevent these events, most commonly intrathecal (IT) chemotherapy. The chemotherapeutic agents approved for intrathecal use in the United States include methotrexate, cytarabine, liposomal cytarabine, and thiotepa [1, 5, 6]. The scheduling and dosing of these medications varies depending on whether they are used for prophylaxis or treatment. Corticosteroids are frequently included with IT chemotherapy, most commonly hydrocortisone, to increase cytotoxicity and to decrease the risk of chemical arachnoiditis [1]. Most prophylactic regimens for leukemia and lymphomas contain methotrexate, either as a single agent or in combination with cytarabine.

The goal of IT chemotherapy is to maximize CNS drug exposure through direct CSF introduction, while reducing systemic drug toxicities [2]. The narrow therapeutic index and high potential toxicities of these agents mean IT administration can have potentially catastrophic consequences. Chemical arachnoiditis, an acute syndrome occurring hours after injection and characterized by headache, backache, vomiting, fever, meningismus, and cerebral fluid pleocytosis, is among the most common and potentially serious effects [1, 7]. More severe symptoms also have been reported including cauda equina syndrome, encephalitis, papilledema, myelopathy, paraplegia, cranial nerve palsies, and seizures [7–11]. It is possible that the incidence of neurological complications in this setting is underestimated because cases may go unrecognized or unreported. To better characterize the incidence of neurological complications secondary to IT therapy, we examined sequential adult patients who received it over a two-year period at our institution. We documented signs and symptoms of neurotoxicity not present before administration that developed acutely thereafter. We provide illustrative case example followed by analysis of events in 109 consecutive patients.

## 2. Methods

We included adult patients with hematologic and solid tumors who received IT chemotherapy between January 2014 and December 2015 at Jackson Memorial Hospital and Sylvester Comprehensive Cancer Center. Primary endpoint studied was development of new symptoms indicative of neurotoxicity and/or arachnoiditis within fourteen days of administration of IT chemotherapy, specifically paralysis, paresthesias, headache, back pain/nuchal rigidity, asthenia, fever, nausea, or vomiting. Additional sensory and sphincter disturbances, which may also be associated with arachnoiditis, were not reliably documented and were excluded from analysis. We defined paralysis and paraesthesias as significant neurologic events for the purposes of analysis and the other side effects as minor events. This division was to allow distinction between more serious neurologic toxicities associated with IT chemotherapy from events with less impact on quality of life and/or of a more systemic nature.

Known CNS involvement was defined as follows: (1) CSF positive for malignancy by cytology and/or flow cytometry from samples collected at the time of administration or previously in association with the patient's current malignancy; (2) contrast enhanced MRI of the brain and/or spinal cord showing leptomeningeal carcinomatosis according to attending radiologist's report [12].

Patient and disease variables were compared between treatment modalities using the chi-square test for categorical data and the Mann-Whitney U test for continuous data. Relative risk was estimated using univariate Poisson regression models to assess the effect of these characteristics on a specific adverse event with respect to the treatment modalities. Tests were two-sided, and findings were considered statistically significant at p<0.05. All analyses were performed using SAS and R software.

This study was a retrospective chart review that did not involve any interaction with patients and therefore specific informed consent from each patient was not required by our Institutional Review Board. Any information that might lead to the identification of individual patients has been excluded. All procedures followed were in accordance with the ethical standards of the responsible committee on human experimentation (institutional and national) and with the Helsinki Declaration of 1975, as revised in 2008.

## 3. Results

### 3.1. Illustrative Case, Severe Neurologic Complications of IT Chemotherapy

A 61-year-old Hispanic woman with a history of stage IV diffuse large B-cell lymphoma (DLBCL) was admitted for salvage therapy with rituximab, dexamethasone, high-dose cytarabine, and cisplatin (R-DHAP). She previously had completed six cycles of rituximab, cyclophosphamide, adriamycin, vincristine, and prednisone (R-CHOP) with twelve milligrams of intrathecal (IT) methotrexate (MTX) prophylactically in each cycle.

On day 1 of R-DHAP the patient received IT MTX 12 mg with cytarabine 50 mg. Flow cytometry and cytology from the lumbar puncture were negative for malignancy. The following day the patient complained of a nonpositional headache rated as 7 out of 10 in intensity. The headache was associated with photophobia, nausea without vomiting, and double vision. She denied neck stiffness or fever. Acetaminophen did not relieve the pain but sumatriptan provided mild relief. On day three she reported bilateral lower extremity weakness, right greater than left. She also reported inability to ambulate secondary to the weakness, rectal incontinence, and urinary retention. Weakness progressed in the days that followed to bilateral lower extremity paralysis.

**Figure 1:**
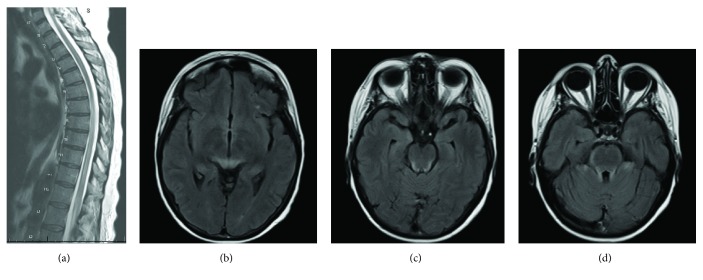
*MRI images of severe IT chemotherapy complications.* (a) Increased T2 signal throughout the spinal cord, most prominent from T6 to the conus. There is some associated cord expansion. The cord signal involves almost the entire diameter most noted at T8. (b-d) Brain images showing symmetric FLAIR signal abnormality in the brainstem, cerebellum, and possibly the thalami without diffusion restriction or abnormal enhancement.

Neurologic exam was significant for right lateral rectus paresis (with the rest of the cranial nerve exam unremarkable), decreased strength of all muscle groups in the bilateral lower extremities, diminished reflexes in the bilateral patellar and Achilles tendons, positive Babinski on the right, and diminished sensation to light touch over the sacrum, posterior thighs, and perineum. The physical exam findings were not present prior to administration of IT chemotherapy. Six days after symptom onset repeat LP again yielded negative cerebrospinal fluid (CSF) studies for involvement by malignant cells. CSF total protein was elevated to 131 mg/dL, glucose and total cell count were within normal limits, and gram stain and culture were negative. Lumbar and thoracic spine MRIs revealed mild enhancement of the ventral and dorsal nerve roots of the cauda equina, particularly at T12-L3, and diffuse central spinal cord signal abnormality most prominent from T6-L2 (Figure 1(a)). Brain magnetic resonance imaging (MRI) showed symmetric FLAIR signal abnormality in the brainstem and cerebellum without diffusion restriction or abnormal enhancement (Figures 1(b)-1(d)).

No other etiology for her symptoms was identified, and they were attributed to intrathecal chemotherapy-induced neurotoxicity. The patient did not recover neurologic function, and her systemic lymphoma unfortunately progressed soon thereafter. She entered hospice care and died secondary to complications of her systemic lymphoma.

### 3.2. Consecutive Case Series

During the study period, 109 patients received IT chemotherapy, of whom 74 (68%) were male and 35 (32%) were female. Forty-four (40%) were Hispanic. The median patient age was 50 years old; the age range was 20 to 88 years old. The most common diagnosis was diffuse large B-cell lymphoma (40%), followed by B-cell ALL (28%), T-cell ALL (8%), and Burkitt lymphoma (8%). Sixteen (15%) of patients were HIV positive and 3 (2.8%) had chronic renal failure. At time of treatment, 33 (30%) patients had CNS involvement (Table 1). The median number of IT chemotherapy doses per patient was 2 (range 1-12).

Therapy consisted of methotrexate alone, cytarabine alone, or methotrexate + cytarabine. Neither thiotepa nor topotecan was used at either institution in adult patients during the time period in question. The total number of IT doses recorded was 355. There were 150 doses of methotrexate alone, 18 of cytarabine alone, and 187 of cytarabine + methotrexate.

**Figure 2:**
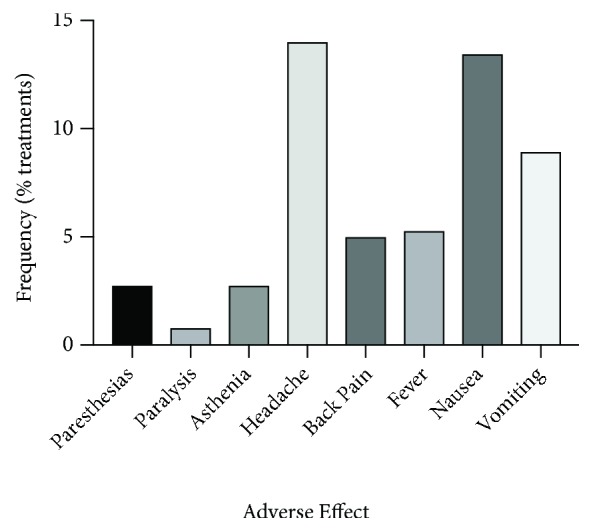
*Rates of adverse effects of IT chemotherapy.* Percentage of 355 IT chemo doses associated with each side effect in a series of 109 consecutive patients.

Rates of each symptom per administration of IT chemotherapy are shown in Table 2 and Figure 2. We also determined the rate at which each symptom occurred per patient over the all doses administered (Table 2). The significant neurologic events paralysis and/or paresthesias occurred after 11 doses (3.10%) affecting 9 patients (8.26%). Minor events occurred after 109 doses (30.70%) affecting 29 patients (26.61%). We documented only new-onset symptoms because the systemic changes fever, nausea, vomiting, and asthenia, which may be associated with chemical arachnoiditis, may also occur for other reasons in this patient population (see Discussion). We compared rates of adverse events among the three treatment modalities but did not detect any significant differences (Table 2). When liposomal cytarabine (Depocyte) was compared to the nonliposomal formulation, there was again no significant difference in rates of adverse neurologic events. There was significant correlation, however, between the number of IT treatments a patient received and likelihood of experiencing at least one adverse effect (correlation coefficient 0.35, p=0.001).

We also examined correlation between adverse neurologic events and independent variables, specifically HIV status, renal failure, and known CNS involvement. Patients with known CNS involvement were found to have risk of any event with borderline statistical significance (RR=2.9, 95%CI=0.99-8.49, p=0.052) but with high significance when considering only minor events (RR=4.35, 95%CI=1.85-10.24, p=0.0008). There were no differences detected in patients who had renal failure or HIV.

## 4. Discussion

Overall incidence of acute neurotoxicity from IT MTX in children is 3-11% [13], but review of the literature reveals rates are not well defined in adults. We report rates of significant adverse neurologic events following IT chemotherapy used as either prophylaxis or treatment for known leptomeningeal involvement at our medical center over a two-year period. We found these events occurred after 3.1% of IT chemotherapy doses, affecting 8.26% of the patients in our consecutive case series. Minor side effects were more common, occurring after 30.70% of doses and affecting 26.61% of patients at least once during the course of their therapy. We found a strong correlation between number of IT treatments received and likelihood of suffering at least one adverse effect. Headache, nausea, vomiting, back pain, and fever were most common—all of which are known symptoms of chemical arachnoiditis. Our results indicate an incidence of neurologic side effects secondary to intrathecal chemotherapy in adults significantly higher than for children and which may be higher than what is commonly perceived by practitioners.

Although symptoms such as headache and back pain related to lumbar puncture are not uncommon, clinicians should also be cognizant that these symptoms may signify impending onset of more significant toxicity. Methotrexate is typically assumed to be the major cause of such neurotoxicities [13], but cytarabine is also a known major cause [14–16]. Our series did not reveal any differences in relative risk of neurologic side effects between MTX, cytarabine, or the two in combination. Jabbour et al. evaluated neurologic complications secondary to IT liposomal cytarabine in combination with high-dose methotrexate as prophylactic treatment in patients with ALL and found the incidence of severe complications to be 16% [8]. A smaller retrospective review by Gállego Pérez-Larraya et al. studying IT liposomal cytarabine given as prophylaxis in patients with non-Hodgkin lymphoma reported 28% of patients developed moderate or severe neurotoxicity [14]. Pretreatment with oral dexamethasone has been found to decrease adverse side effects of depot form of cytarabine (DTC) [17].

IT chemotherapy for both therapy and prophylaxis of CNS involvement has been a mainstay for medical management of leukemia and lymphoma throughout the world for several decades and for patients with leptomeningeal involvement by solid tumors [7]. Survival for patients with leptomeningeal spread of disease, however, is low while the incidence of early and late complications associated with IT chemotherapy can be high [18–20]. Several studies have suggested that systemic high-dose (HD) MTX may improve the response rate or survival of patients with CNS involvement [18, 19]. Glantz et al. treated 16 patients with neoplastic meningitis from a variety of solid tumors and lymphoma with HD MTX alone and retrospectively compared outcomes to 15 patients treated with standard IT chemotherapy [19]. Significantly longer median survival was found in patients who received HD IV MTX (13.8 months) compared to those who received IT MTX (2.3 months; P=0.003). Daily CSF samples were collected and MTX concentrations were measured, revealing that IV administration achieved superior CSF MTX levels. As the flow of CSF is from the ependymal cells in the brain down to the cauda equina where it is resorbed, the administration of IT MTX to the area between L2 and L3 was suboptimal. IT topotecan has been used with some success in pediatric malignancies based on favorable pharmacokinetic properties [21, 22]. In adults, however, IT topotecan as a single agent did not produce clinical benefit over standard therapies in a multicenter phase 2 trial for patients with meningeal involvement by any malignancy [23]. A later case series, however, showed anecdotally particular patients may achieve clinical benefit lasting up to 12 months from IT topotecan [24].

Olmos-Jimenez et al. performed an observational and prospective study in Spain evaluating standardized triple intrathecal chemotherapy in adult hematology-oncology patients over an 18-month period [25]. Similar to our study, adverse events occurring after administration of IT chemotherapy was recorded; however this study was substantially smaller containing only 20 patients and 56 treatments. The study population was 75% male, 50% of the patients had non-Hodgkin lymphoma, and 5% had pre-existing leptomeningeal disease. Adverse events occurred after 39.3% of 56 doses recorded. The vast majority of events (96.7%) were grades 1-2 with only one event being grade 3. As in our study, the adverse event recorded most frequently was headache, followed by vomiting and vertigo. One administration event (1.8%) resulted in grade 2 sensorimotor polyneuropathy.

## 5. Limitations

Because our study was retrospective, the ability to determine causality of symptoms is limited and we acknowledge that the population studied had additional reasons to develop some of the symptoms reported. Given the disparate underlying malignancies of the patients in our series, we did not investigate systemic chemotherapy as a confounder; future studies should investigate whether concurrent administration of agents that may cause neuropathy, such as vincristine, may lead to increased rates of neurologic events with administration of IT chemotherapy. We believe for the great majority of incidents we analyzed here, however, the timing of the symptoms in relation to IT chemotherapy administration reflects a likely causative link. An additional caveat of this study is that we could not specifically separate out prophylactic measures such as administration of hydrocortisone as independent variables in our analysis due to low numbers and incomplete documentation of nonpharmacologic interventions. Questions regarding efficacy of interventions designed to prevent IT chemo toxicities are likely best answered through prospective studies. Use of the Common Toxicity Criteria (CTC) to define symptoms was considered and would have made the side effects we report more easily comparable to experiences at other institutions. This was not possible for the purposes of this retrospective analysis, however, in which patient charting did not consistently follow the criteria. This is another matter for which a prospective study would be well suited.

## 6. Conclusion

The most common approach for prevention of CNS spread of hematologic tumors is IT chemotherapy, with or without radiation. Its use should not be discarded, but practitioners should be aware of the potential complications and a frequency that may be higher than commonly perceived. Consideration of alternate, less toxic forms of therapy such as systemic HD MTX may be warranted and, as highlighted, could be more effective in some cases.

## Figures and Tables

**Table 1:** Patient population and disease characteristics.

Variable	N	%
Gender		
Female	35	32.1
Male	74	67.9
Race/Ethnicity		
Non-Hispanic White	20	18.3
Non-Hispanic Black	17	15.6
Hispanic White	42	38.5
Hispanic Black	2	1.8
Asian	2	1.8
Haitian	4	3.7
Other/unknown	22	20.2
Type of Cancer		
DLBCL	43	39.4
B-Cell ALL	31	28.4
T-Cell ALL	8	7.3
Burkitt Lymphoma	8	7.3
Breast Cancer	3	2.8
Other Non-Hodgkin	7	6.3
CML	1	0.9
CLL	1	0.9
Other	4	3.7
Chronic Renal Failure		
Yes	3	2.8
No	106	97.2
HIV		
Yes	16	14.7
No	93	85.3
CNS Involvement		
Yes	33	30.3
No	76	69.7

**Table 2:** Adverse neurologic events.

	No. of patients affected (%)	No. of Events ( occurrence rate per IT treatment)	Treatment	RR	95% CI	P Value
Significant Neuro Events	9 (8.26)	11 (3.10)				

Overall*∗*			MTX	1	-	-
			CYT	1.67	0.19-14.27	0.641
			MTX and CYT	1.16	0.50-2.71	0.728

Paresthesias	8 (7.34)	10 (2.82)				

			MTX	1	-	-
			CYT	1.67	0.19-14.27	0.64
			MTX and CYT	0.64	0.17-2.39	0.51

Paralysis*∗∗*	2 (1.83)	3 (0.85)				

			MTX	1	-	-
			CYT	8.33	0.52-133.23	0.134
			MTX and CYT	0.80	0.05-12.82	0.876

Minor Events	29 (26.61)	109 (30.70)				

Overall*∗*			MTX	1	-	-
			CYT	1.53	0.75-3.12	0.241
			MTX and CYT	0.97	0.66-1.41	0.857

Asthenia	4 (3.67)	10 (2.82)				

			MTX	1	-	-
			CYT	2.78	0.56-13.76	0.21
			MTX and CYT	0.80	0.26-2.49	0.703

Headache	15 (13.76)	50 (14.08)				

			MTX	1	-	-
			CYT	1.23	0.43-3.53	0.694
			MTX and CYT	0.68	0.39-1.19	0.180

Back pain	9 (8.26)	18 (5.07)				

			MTX	1	-	-
			CYT	2.08	0.44-9.81	0.353
			MTX and CYT	1.00	0.40-2.54	0.996

Fever	5 (4.59)	19 (5.35)				

			MTX	1	-	-
			CYT	2.78	0.56-13.78	0.211
			MTX and CYT	1.47	0.54-3.98	0.447

Nausea	13 (11.93)	48 (13.52)				

			MTX	1	-	-
			CYT	0.93	0.21-3.99	0.918
			MTX and CYT	1.25	0.69-2.26	0.464

Vomiting	6 (5.50)	32 (9.01)				

			MTX	1	-	-
			CYT	1.11	0.25-4.86	0.889
			MTX and CYT	0.91	0.45-1.82	0.788

*∗*Multiple major or minor events simultaneously are counted only once in overall (overall numbers are therefore less than total of subcategories).

*∗∗*One patient had both paresthesias and paralysis in two events. To avoid double-counting, these events were included only in paresthesias for the RR calculation.

## Data Availability

The pooled statistical data used to support the findings of this study are included within the article. Additional details used to support the findings of this study are available from the corresponding author upon request, but we cannot release data that could lead to identification of specific patients.
